# Biological Characteristics and Pathogenicity Analysis of a Low Virulence G2a Porcine Epidemic Diarrhea Virus

**DOI:** 10.1128/spectrum.04535-22

**Published:** 2023-05-18

**Authors:** Kechen Yu, Xueting Liu, Ying Lu, Meijing Long, Jiaguo Bai, Qiuying Qin, Xueli Su, Guifu He, Xue Mi, Chunjie Yang, Ruomu Wang, Hejie Wang, Ying Chen, Zuzhang Wei, Weijian Huang, Kang Ouyang

**Affiliations:** a Guangxi Colleges and Universities Key Laboratory of Prevention and Control for Animal Disease, College of Animal Science and Technology, Guangxi University, Nanning, China; b Guangxi Zhuang Autonomous Region Engineering Research Center of Veterinary Biologics, Nanning, China; c Guangxi Key Laboratory of Animal Reproduction, Breeding and Disease Control, Nanning, China; University of Manitoba

**Keywords:** porcine epidemic diarrhea virus, virus isolation, low virulence, molecular characteristics, pathogenicity

## Abstract

Since the outbreak caused by a porcine epidemic diarrhea virus (PEDV) variant in 2010, the current epidemic of PEDV genotype 2 (G2) has caused huge economic losses to the pig industry in China. In order to better understand the biological characteristics and pathogenicity of the current PEDV field strains, 12 PEDV isolates were collected and plaque purified during 2017 to 2018 in Guangxi, China. The neutralizing epitopes of the spike proteins and the ORF3 proteins were analyzed to evaluate genetic variations, and they were compared with the reported G2a and G2b strains. Phylogenetic analysis of the S protein showed that the 12 isolates were clustered into the G2 subgroup (with 5 and 7 strains in G2a and G2b, respectively) and that they shared 97.4 to 99.9% amino acid identities. Among them, one of the G2a strains, CH/GXNN-1/2018, which had a titer of 10^6.15^ PFU/mL, was selected for pathogenicity analysis. Although piglets infected with the CH/GXNN-1/2018 strain exhibited severe clinical signs and the highest level of virus shedding within 24 h postinfection (hpi), recovery and decreased virus shedding were seen after 48 hpi, and no piglets died during the whole process. Thus, the CH/GXNN-1/2018 strain had low virulence in suckling piglets. Virus neutralizing antibody analysis showed that the CH/GXNN-1/2018 strain induced cross-protection against both homologous G2a and heterologous G2b PEDV strains as early as 72 hpi. These results are of great significance for understanding PEDV in Guangxi, China, and they provide a promising naturally occurring low-virulent vaccine candidate for further study.

**IMPORTANCE** The current epidemic of porcine epidemic diarrhea virus (PEDV) G2 has caused huge economic losses to the pig industry. Evaluation for low virulence of the PEDV strains of subgroup G2a would be useful for the future development of effective vaccines. In this study, 12 field strains of PEDV were obtained successfully, and they were characterized from Guangxi, China. The neutralizing epitopes of the spike proteins and the ORF3 proteins were analyzed to evaluate antigenic variations. One of the G2a strains, CH/GXNN-1/2018, was selected for pathogenicity analysis, and it showed that the CH/GXNN-1/2018 strain had low virulence in suckling piglets. These results provide a promising naturally occurring low-virulent vaccine candidate for further study.

## INTRODUCTION

Porcine epidemic diarrhea (PED) is an acute intestinal infectious disease of pigs caused by the PED virus (PEDV), which is clinically manifested as watery diarrhea and severe dehydration (1, 2). The morbidity rate in suckling piglets is up to 80 to ~100% (3), and it is one of the major diseases which causes the early death of piglets. The disease was first reported in the United Kingdom in 1976 (4). PEDV genotype 2 (G2) has caused outbreaks in China, Thailand, and other Asian countries in 2010 (5). In 2013, a large-scale epidemic form of PED occurred in the United States, causing huge economic losses to the pig industry (6).

PEDV is a coronavirus and it belongs to the genus *Alphacoronavirus* within the *Coronaviridae* family, *Alphacoronavirus*. It is an enveloped, single-stranded, positive-sense RNA virus. PEDVs are shaped as pleomorphic and are mainly spherical, with diameters ranging from 95 nm to 190 nm. The genome size of PEDV is approximately 28 kb (7), and it possesses untranslated regions (UTRs) at both ends and at least seven open reading frames (ORFs). It contains four structural proteins, including spike (S), envelope (E), matrix (M), and nucleocapsid (N) proteins (8). The S protein in PEDV plays an important role in specific receptor binding, cell entry, and stimulating the body to produce neutralizing antibodies (9). Five neutralizing epitope regions have been identified, including the N-terminal domain S10 region (NTD/S10), the collagenase equivalent domain (COE), the SS2 and SS6, and the C-terminal epitope 2C10 (10, 11). Moreover, the S gene is used frequently to evaluate the genetic diversity of PEDVs.

The PEDVs are comprised of genotype 1 (G1) and 2 (G2) based on the S gene, and G2 has been further classified into G2a, G2b, and G2c subtypes (12). The subgroups G2a and G2b are currently prevalent in nearly all of the PEDV variations in China and are highly pathogenic to suckling piglets (5, 12, 13). However, low virulence PEDV strains belonging to the G2 subgroup have also been reported. The piglets infected with the JS-A strain of the G2a subgroup showed only microlesions in the jejunum and ileum, and no piglets died throughout the experiments carried out (14). The HLJBY strain of the G2b subgroup showed a low pathogenicity similar to that of the vaccine strain CV777 (15). Additionally, the PEDVs of the G2a subgroup provide better immune protection against the homologous and heterologous PEDV challenges than the G2b subgroup strain (12). Evaluation for low virulence of the PEDV strains of subgroup G2a would be useful for the future development of effective vaccines.

Recently, numerous studies have demonstrated that commercially available PEDV vaccinations derived from classical PEDV strains are ineffective against the PEDV variant infections in China. This issue has resulted in substantial morbidity and death in neonatal piglets (6, 16, 17). Therefore, there is an urgent need to develop new vaccines based on the PEDV variations. In order to achieve this objective, we isolated and purified the PEDV variant strain CH/GXNN-1/2018 from the presently widespread Chinese genotype G2a subgroup. In this study, we investigated its biological characteristics and pathological capabilities with a view of producing a more effective vaccine against PEDV.

## RESULTS

### Viral isolation and identification.

**FIG 1:**
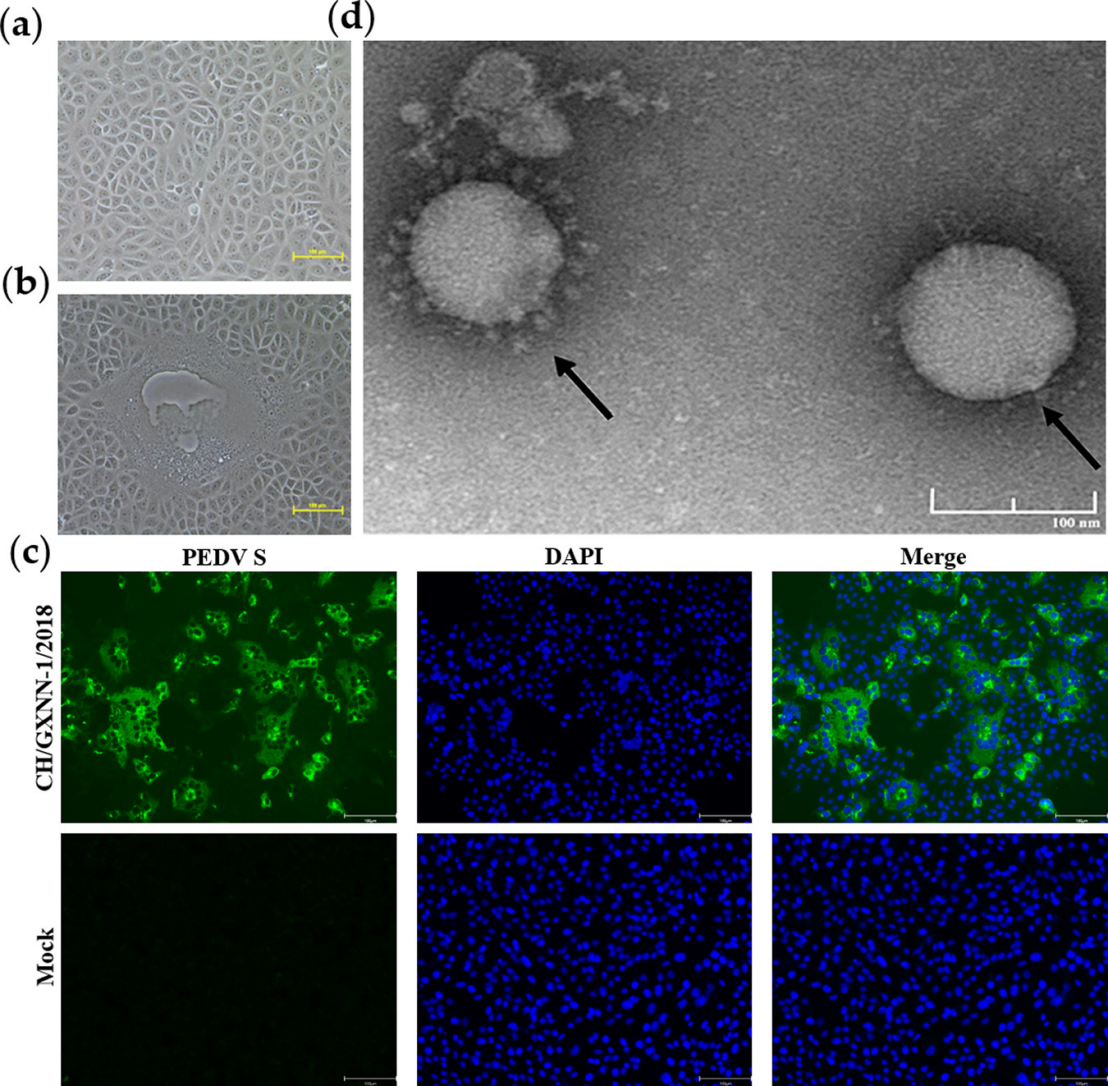
Isolation and identification of a representative PEDV strain CH/GXNN-1/2018 in Vero cells. (a) Control (uninfected) Vero cells (200×). (b) CH/GXNN-1/2018-inoculated Vero cells at 48 hpi showing rounded and clustered cells (200×). (c) Detection of PEDV strain CH/GXNN-1/2018 in Vero cells by immunofluorescence assay at 72 hpi (200×); anti-PEDV S protein monoclonal antibody and fluorescein isothiocyanate (FITC)-conjugated goat anti-mouse immunoglobulin G (IgG) were used as primary and secondary antibodies, respectively. (d) Electron microscopy images of PEDV virions from cell culture media of Vero cells infected with CH/GXNN-1/2018 (Magnification, ×80,000).

In this study, the cytopathic effects (CPEs) as characterized by cell fusion, syncytial, and vacuole formation from 12 samples, were observed, and they were confirmed as PEDV positive by using reverse transcriptase PCR (RT-PCR) targeting the M gene. Furthermore, viral propagation was confirmed by the detection of PEDV antigens with immunofluorescence assay (IFA) using an anti-PEDV S protein monoclonal antibody. Specific green fluorescence was visible in Vero cells at 72 hpi, but no fluorescence was detected in control cultures. Transmission electron microscopy (TEM) showed that the virions were round and about 100 nm in diameter, with surface petal-like fibrils that are classical characteristics of coronaviruses. The viral isolation and identification of a representative strain CH/GXNN-1/2018 were shown in Fig. 1.

### Propagation of PEDV strains in Vero cells.

After identification, there were 12 strains with typical CPEs and positive assessment by PCR, which were from the cities of Nanning, Liuzhou, and Beihai (Table 1). All of them were isolated from samples of small intestine contents (SICs). RNA titers for these 12 PEDV strains in Vero cells were determined by reverse transcriptase quantitative PCR (RT-qPCR) (18). The results showed that 12 isolates propagated in Vero cells with 5.37 to 8.87 log_10_ genomic equivalents (GE)/mL.

**TABLE 1:** A summary of the PEDV strains isolated in Vero cells

Isolate name	Collection date (yr, mo)	Collection area	Specimen	PEDV titer (log_10_ GE/mL)	GenBank accession no.
CH/GXLZ-1/2017	2017.02	Liuzhou	SIC	7.56	MZ669841
CH/GXBH-1/2018	2018.03	Beihai	SIC	7.59	MZ669844
CH/GXBH-2/2018	2018.03	Beihai	SIC	5.37	MZ669845
CH/GXNN-1/2018	2018.03	Nanning	SIC	7.30	MZ669834
CH/GXNN-2/2018	2018.03	Nanning	SIC	6.88	MZ669835
CH/GXNN-3/2018	2018.04	Nanning	SIC	8.68	MZ669836
CH/GXNN-4/2018	2018.04	Nanning	SIC	8.61	MZ669837
CH/GXLZ-2/2018	2018.04	Liuzhou	SIC	7.69	MZ669842
CH/GXLZ-3/2018	2018.04	Liuzhou	SIC	8.87	MZ669843
CH/GXNN-5/2018	2018.05	Nanning	SIC	8.54	MZ669838
CH/GXNN-6/2018	2018.07	Nanning	SIC	8.66	MZ669839
CH/GXNN-7/2018	2018.07	Nanning	SIC	8.63	MZ669840

### The alignment of amino acid sequences.

**FIG 2:**
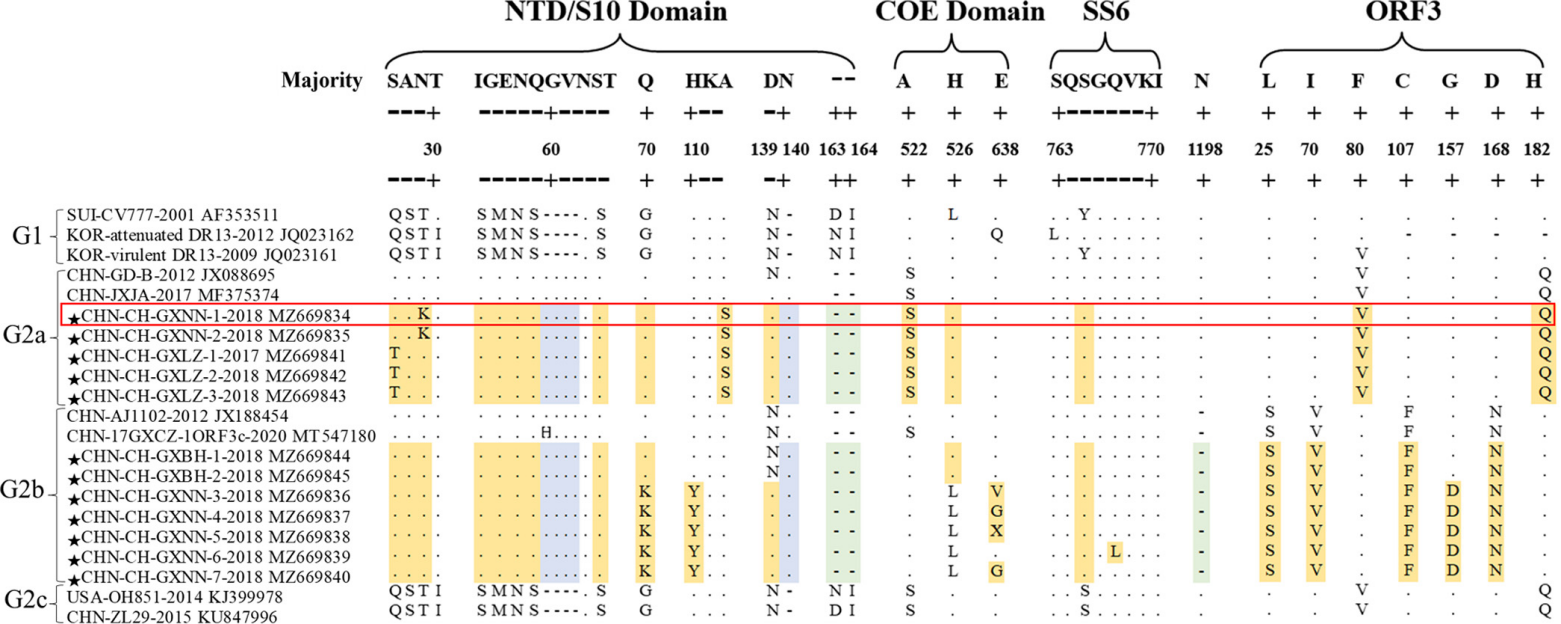
Amino acid variations in the S and ORF3 protein between the different subgroups of PEDV. S and ORF3 protein amino acid sequences of 12 isolates, including the CH/GXNN-1/2018 strain (marked with the red box) and other reference PEDV strains were aligned using Clustal W. The dots and the cross-lines represent the consensus and deleted amino acids, respectively. The regions inserted, deleted, and substituted relative to CV777 are in blue, green, and yellow highlights, respectively.

The pairwise alignment analyses revealed multiple amino acid insertions, deletions, and mutations (Fig. 2). Five epitopes (NTD/S10, COE, SS2, SS6, and 2C10) were analyzed to evaluate antigenic variations. All the detected strains were generally conserved in epitopes SS2 and 2C10, while typical variation patterns were observed in the NTD/S10, COE, and SS6 domains. It is noteworthy that the 5 isolates belonging to the G2a subgroup in this study contained 3 additional single mutations at S27T, N29K, and A112S in the NTD/S10 epitope compared with other reported G2a strains (GD-B and JXJA). Moreover, 5 of the 7 isolates in the G2b subgroup had 4 additional mutations at Q70K, H110Y, N139D, and H526L in the NTD/S10 and COE epitopes compared with other reported G2b strains (AJ1102 and 17GXCZ-1ORF3c), and a 1-amino-acid (N) deletion was observed at position 1198 in all the G2b isolates.

In addition, to further understand the genetic diversity of PEDVs in Guangxi, the amino acid sequences of ORF3 proteins of these 12 isolates were compared. The results showed that 5 of the 7 isolates in subgroup G2b had a unique mutation at G157D, while other isolates of the ORF3 protein were conserved between the same subgroups.

### Phylogenetic analysis of the S protein.

**FIG 3:**
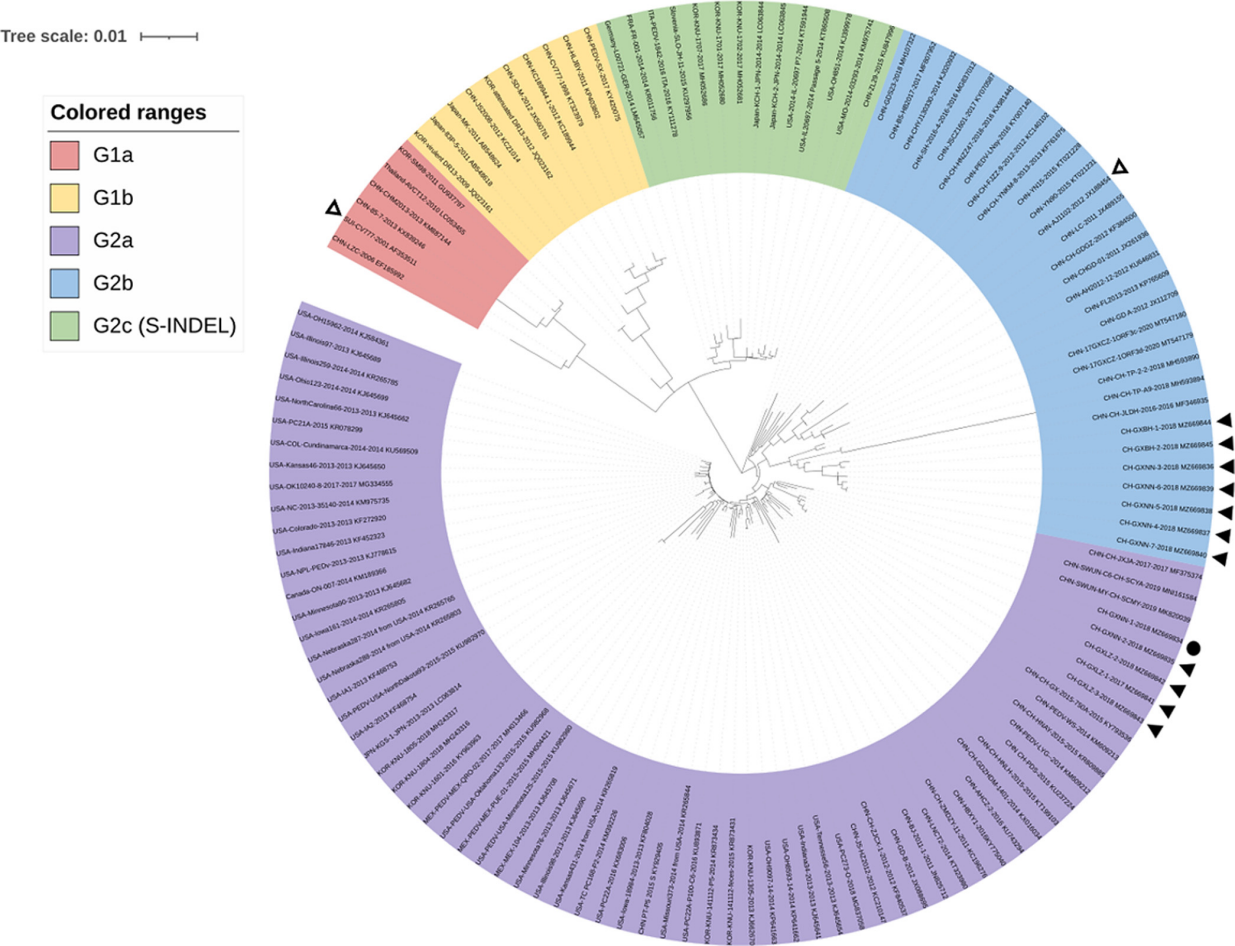
Phylogenetic analysis based on the S protein of our 12 isolates and the 118 historic strains available from the GenBank. Phylogenetic trees were constructed by using the neighbor-joining method from MEGA 5.2, with 1,000 bootstrap replicates. The scale bar represents the branch lengths as measured by the number of substitutions per site. The reference PEDV strains are indicated in the following format: country of origin (three-letter code: CHN, China; JPN, Japan; KOR, South Korea; MEX, Mexico; SUI, Switzerland; Ger, German; Fra, France; Ita, Italy and USA, the United States)/strain name/year of sample collection (GenBank accession number). Subgroups G1a, 1b, 2a, 2b, and 2c are shown in red, yellow, purple, blue, and green, respectively. The solid circular symbol represents the strain CH/GXNN-1/2018, and the solid triangle symbols represent the other 11 strains obtained in this study. The hollow triangle symbols represent the vaccine strains CV777 and AJ1102.

The full-length S genomes of the 12 isolates were determined to be 4,158 to 4,161 nucleotides (nt) (GenBank accession numbers MZ669834-MZ669845). Furthermore, the S protein of the 12 isolates shared 97.4 to 99.9% amino acid identities. A phylogenetic tree was constructed based on the S protein of the 12 isolates and another 118 reference strains that were available from GenBank (Fig. 3). Our 12 isolates were all distributed into G2 with 5 strains, including CH/GXNN-1/2018 clustered in G2a and 7 other strains were in G2b. The 12 isolates were clustered closely around the Chinese isolates, such as the AJ1102 (JX188454) strain, but they were distant from both the North America epidemic and Europe strains. The strain CH/GXNN-1/2018 (MZ669834) clustered into G2a and was distant from the Belgium-CV777 (AF353511) and the attenuated DR13 (JQ023162) vaccine strains.

### Determination of viral titers.

**FIG 4:**
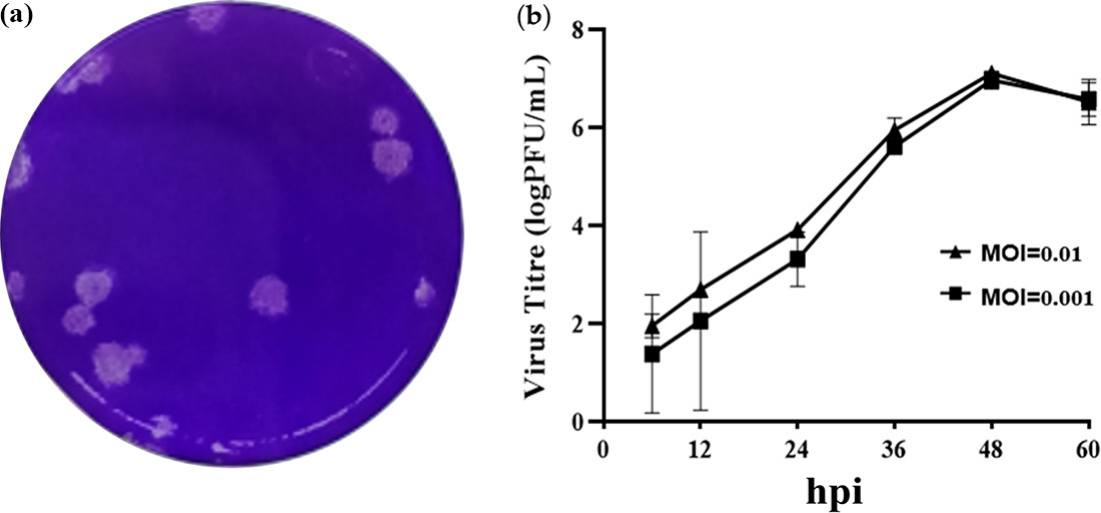
Plaque purification and viral growth kinetics of the PEDV strain CH/GXNN-1/2018. (a) Purification of the PEDV strain. (b) Viral growth kinetics of CH/GXNN-1/2018 in Vero cells. The PEDV strain CH/GXNN-1/2018 was propagated in Vero cells at an MOI of 0.01/0.001, which was determined at different time points, and the PEDV titers were calculated (log PFU/mL).

The strain CH/GXNN-1/2018, which belongs to subgroup G2a, was selected for further investigation of biological characterization and pathogenicity evaluation. The plaques from CH/GXNN-1/2018 showed a rounded and unitary shape, and a culture of the strain had a titer of 10^6.15^ PFU/mL (Fig. 4a). When the isolated strain CH/GXNN-1/2018 was inoculated into Vero cell cultures at a multiplicity of infection (MOI) of 0.01 or 0.001, the trends of the viral replication and proliferation curves were the same for the multistep growth curves. We noted that the virus started to replicate at 6 h, reaching a peak at 48 h, and then decreased thereafter (Fig. 4b).

### Pathogenicity analysis of the CH/GXNN-1/2018 strain.

**FIG 5:**
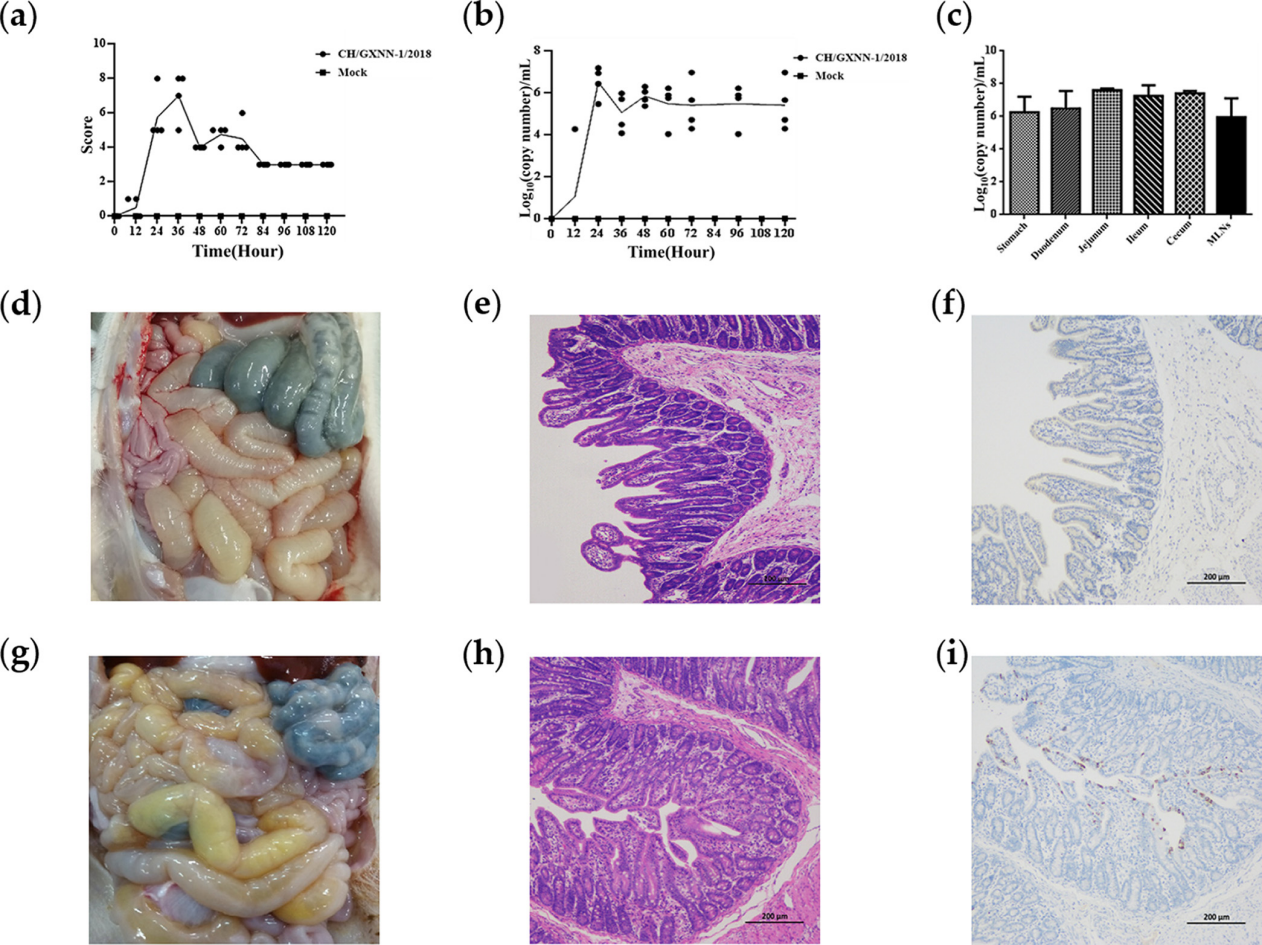
The replication and pathogenesis of the CH/GXNN-1/2018 strain in piglets. (a) Clinical signs included the following five assessment parameters: appetite status, mental status, hair condition, diarrhea, and vomiting (scores for each assessment were 0 = normal, 1 = slight change, 2 = obvious change, and 3 = severe change). The total score for each piglet was added up by each assessment score (the range of accumulated scores from 0 to 15 for each piglet). (b) Virus shedding in the feces. The viral load was taken and assessed by real-time PCR. (c) Qualification of viral abundance in different intestinal tissues. The piglets were necropsied upon death, whereas all surviving piglets from the challenged and mock groups were euthanized at 5 dpi for postmortem examinations. The solid dot and solid square indicate the clinical scores (a) and the PEDV copy number (b) in the rectal swab from one piglet in challenge or mock group. Necropsy examinations and histopathological and immunohistochemical (IHC) staining were performed of the intestines of piglets inoculated with the CH/GXNN-1/2018 strain. Different tissue samples, including the duodenum, jejunum, ileum, cecum, mesenteric lymph nodes, and stomach, from each group were taken and then they were processed for H&E and IHC staining. Representative images of the jejunum are shown (100×). (d, e, and f) Show the results of necropsy examinations, IHC, and H&E staining images of the control group, respectively. (g) The small intestines of the CH/GXNN-1/2018-inoculated group were distended, transparent, and filled with yellow fluid. (h) The jejunum from the CH/GXNN-1/2018-inoculated piglets was characterized by the shortening, atrophy, and shedding of the intestinal villi. (i) PEDV antigen appears as a brown stain and was detected in the epithelial cells of the jejunum in the CH/GXNN-1/2018-inoculated piglets. The intestinal tissue sections were stained with an anti-PEDV spike protein monoclonal antibody followed by incubation with an HRP-conjugated goat anti-mouse antibody and then visualized using a fluorescence microscope.

To investigate the pathogenicity characteristics of the CH/GXNN-1/2018 strain, we randomly divided eight 7-day-old piglets into two groups and orally inoculated them with the CH/GXNN-1/2018 strain at a dose of 6 log_10_ PFU/mL (2 mL/piglet) and Dulbecco’s modified Eagle medium (DMEM; 2 mL/piglet), respectively. During the challenge period, the piglets in the CH/GXNN-1/2018 group started to have diarrhea at 12 hpi, and the most severe symptoms were accompanied by vomiting and dehydration at 24 hpi. However, the symptoms decreased and the physiological state recovered after 48 hpi, and no piglets died during the challenge period (Fig. 5a). All the piglets in the control group were physiologically fine and did not show any of the above clinical symptoms.

We determined viral shedding in the feces by using RT-qPCR that targeted the PEDV M gene. After 12 hpi, PEDVs were detected in all the rectal swabs of the four piglets in the experimental group with a 100% infection rate. At 24 hpi, viral shedding reached its maximum and then it stabilized by the end of the experiment (Fig. 5a). All the piglets in the control group were negative for PEDV. Viral loads were slightly higher in the jejunum than those the other intestinal tissues, and the PEDVs were also detected in the mesenteric lymph nodes (MLNs), stomachs, duodenum, ileum, and cecum (Fig. 5a).

The piglets challenged with the CH/GXNN-1/2018 strain showed gross lesions macroscopically. The whole small intestines swelled with a yellowish watery fluid, and the intestinal walls appeared thin and transparent. Histopathological analysis indicated severe shrinkage and fusion of small intestinal villi and vacuolization of the epithelial cells. Immunohistochemistry (IHC) staining showed that the PEDV antigen was distributed mainly in the cytoplasm of epithelial cells of the atrophied villi (Fig. 5b). Neither macroscopic nor microscopic intestinal changes nor viral antigens were present in the negative-control piglets.

### The VN antibodies against both G2a and G2b PEDVs.

To determine how effective the neutralizing antibodies produced by the PEDV strain CH/GXNN-1/2018 isolated in this study are at cross-protecting against other G2 subgroup PEDV strains, the virus neutralization (VN) antibodies against both the homologous G2a strain and heterologous G2b strain were determined by VN test. In the experimental group, the homologous VN antibodies were detected as early as 72 hpi (4/4) with the highest VN titers of 1:8. Similarly, the heterologous VN antibodies against G2b PEDV could be detected at 72 hpi (2/4) (Table 2). The serums collected from the mock group were VN antibodies negative for both G2a and G2b strains throughout the study period.

**TABLE 2:** Virus neutralization antibody testing of antisera against G2a and G2b PEDVs^*a*^

Time postinfection (h)	G2a PEDV					G2b PEDV				
	Neutralizing antibody response (titer) of^*b*^:				Total no. of animals	Neutralizing antibody response (titer) of^*c*^:				Total no. of animals
	Pig5	Pig6	Pig7	Pig8		Pig5	Pig6	Pig7	Pig8	
0	−^*c*^	−	−	−	0/4	−	−	−	−	0/4
24	−	−	−	−	0/4	−	−	−	−	0/4
72	+(1:4)	+(1:4)	+(1:4)	+(1:8)	4/4	−	+(1:4)	−	+(1:4)	2/4
120	+(1:4)	−	+(1:8)	+(1:8)	3/4	−	+(1:8)	+(1:4)	−	2/4

a −, indicates that no neutralizing antibody was produced; +, indicates that neutralizing antibody was produced.

b The neutralizing activity of G2a PEDV was evaluated in Vero cells using the parental G2a PEDV CH/GXNN-1/2018 isolate.

c The neutralizing activity of G2b PEDV was evaluated in Vero cells using the heterologous G2b PEDV 17GXCZ-1ORF3d isolate.

## DISCUSSION

In recent years, PEDV has emerged as one of the most lethal and infectious pathogens in pigs, and it has caused significant economic losses on the global pig industry. Biosecurity, vaccination, and feedback are basic approaches to managing the disease, but these approaches are not effective at eradicating the spread of PEDV (19–21). At present, there is still a degree of challenge for the isolation of PEDVs. In this study, we attempted virus isolation using 116 PEDV-positive samples from small intestine contents (SICs), rectal swabs, and feces, but only 12 isolates were obtained from the samples obtained from the SICs. This result indicates that using SIC sampling should be the preferred choice for PEDV isolation. In addition, trypsin is one of the important conditions for the intracellular proliferation of PEDV in Vero cells (22), and we chose a concentration of 15 μg/mL to facilitate viral proliferation and adaptation in these cells.

PEDV has evolved into two separate groups, namely, G1 (classical) and G2 (variant), based on the S genome sequence, and most of the predominant strains after 2010 have been attributed to the G2 group. This group was further subdivided into the G2a and G2b subgroups, and multiple recombinants and S-INDEL strains from around the world can be clustered into a new G2c subgroup by other proposed systems (23, 24). In this study, the 12 isolates all belonged to the G2 group (5 strains in G2a and 7 strains in G2b). Although the collection time of these strains was relative long from the present time, they could still represent the current PEDV field strains to some extent.

The genetic data indicated that the Guangxi isolates were still most closely related to the G2a and G2b pandemic strains but that they continued to evolve independently through the process of genetic drift. Changes in the antigenic epitope amino acids may alter the immunogenicity of the virus (25), which can play an important role in the prevention and control of PED. In the present study, the CH/GXNN-1/2018 strain which belongs to G2a was found to be from a different branch than the vaccine and the earlier epidemic strains from China. It was shown to be a distant relative of these strains. Compared with other reported G2a and G2b strains, it was found that the 12 strains isolated in this study had 7 unique amino acid mutations at S27T, N29K, A112S, Q70K, H110Y, N139D, and H526L in the NTD/S10 and COE epitopes of the S protein and a unique mutation at G157D in the ORF3 protein, separately. Thus, the deletion, insertion, and mutation of the S gene may affect the amino acid sequence, thereby affecting the protein structure and indirectly affecting the virulence of PEDVs. Whether these amino acid substitutions influenced the antigenicity and pathogenicity of PEDVs remains to be investigated.

Previous reports have confirmed that the PEDV strains of the subgroups G2a and G2b are highly pathogenic to suckling piglets, with infections manifesting as severe clinical symptoms, such as watery diarrhea, vomiting, and dehydration. In addition, high viral loads are shed in the stool and severe lesions appear in the intestines (26, 27). Although the viral loads were also notably high in the stomachs and MLNs of the piglets infected with strain CH/GXNN-1/2018 in our study, this finding was inconsistent with the results reported by Yang et al. (17). Our pathogenicity test results revealed that the 7-day-old piglets infected with CH/GXNN-1/2018 had only temporary diarrhea and fecal shedding of high titers of viral RNA at 24 hpi and showed a trend to recover after 36 hpi. There were also no dead piglets during our whole experiment. Our results were similar to previous studies by Huan et al. (15). They isolated a G2b genotype PEDV strain (HLJBY) and orally inoculated newborn piglets without colostrum at a dose of 10^7.0^ 50% tissue culture infective dose (TCID_50_), and diarrhea appeared at 48 h. The diarrhea symptoms lasted until the end of the experiment, but no piglets died during their experiments. Qian et al. (14) also isolated a G2a PEDV strain (JS-A) and orally inoculated 5-day-old newborn piglets at a dose of 3 × 10^5.0^ TCID_50_, and the infection did not cause the death of suckling piglets. Thus, we considered that the CH/GXNN-1/2018 strain had low virulence in suckling piglets. However, it is still difficult to evaluate the pathogenicity of this PEDV strain without the comparison groups of other PEDV strains, and we consider it in our future studies.

Currently, it is thought that the pathogenicity of PEDVs is affected by multigene events. Wang et al. (28) constructed a chimeric infectious clone of the S gene between the highly virulent BJ2011 and the virulent CHM2013 strains, and they showed that the S gene was not the only factor impacting on the virulence of the virus. Lu et al. (29) isolated two G2b genotypes of the PEDV strains that showed >99% nucleotide identity in the S regions but only demonstrated low nucleotide identities (80.5%) in the ORF3 gene. The pathogenicity results indicated that the strains with the truncated ORF3 gene had higher virulence. The factors responsible for these contradictions have not been identified, but virus source, infectious dose, innate immune response, animal/environmental conditions, and nucleotide/amino acid variations between strains might be among the causes (30). In addition, the pathogenicity of PEDVs is also related to the age of the piglets which are infected.

In summary, 12 PEDV isolates were obtained successfully, and they were characterized from Guangxi, China. In these isolates, the epitopes of the S protein showed multiple variations. This study showed that CH/GXNN-1/2018 is a low pathogenic strain that belongs to the G2a subgroup, and it might be a promising PEDV vaccine candidate strain for further study. Our study increased the number of isolates and the pathogenic data of PEDVs. It has also laid the foundation for knowledge regarding the epidemic degree of PEDVs in Guangxi, China, and has contributed to providing reference materials for studying the molecular mechanism of the pathogenicity of viruses.

## MATERIALS AND METHODS

### PEDV diagnosis and clinical sample collection.

Total RNA was extracted using a viral DNA/RNA kit (Axygen Scientific, Union City, CA) and transcribed into cDNA using oligonucleotide dT primers, dNTP mix, and a Moloney-MLV (M-MLV) reverse transcriptase reagent (TaKaRa, Dalian, China). The presence of PEDV was confirmed by using reverse transcription-PCR (RT-PCR) with primers to amplify approximately 492-bp sequences of the N gene as described by Kim et al. (31) (PED-NF [5′-GAA ATAACCAGGGTCGTGGA-3′] and PED-NR [5′-GCTCACGAACAGCCACATTA-3′]).

From January 2017 to May 2019, 116 PEDV-positive samples were assessed. They were small intestine contents (SICs), rectal swabs, and feces samples which were collected from 9 cities in Guangxi. All the samples were diluted with phosphate-buffered saline (PBS) at a ratio of 1:4 and centrifuged at 12,000 rpm at 4°C for 5 min. The supernatants were filtered through a 0.22-μm-pore-size filter and then they were stored at −80°C.

### PEDV isolation.

The PEDVs were isolated in Vero cells as described previously (29). Vero cells were cultured and maintained in Dulbecco’s modified Eagle medium (DMEM; Life Technologies, Carlsbad, CA) supplemented with 10% fetal bovine serum (Biological Industries, Kibbutz Beit Haemek, Israel) and inoculated into 6-well plates at 37°C in an atmosphere of 5% CO_2_. The Vero cell monolayers were washed twice with PBS and then they were inoculated with 100 μL of the 5-fold diluted samples at 37°C. After 1 h of incubation, the cells were maintained in DMEM containing 15 μg/mL trypsin at 37°C in a 5% CO_2_ atmosphere until cytopathic effects (CPEs) became visible. When the CPEs exceeded 80%, the plates were subjected to three freeze-thaw cycles at −80°C. The mixtures were centrifuged at 3,000 × *g* for 5 min at 4°C. The supernatants were either harvested for further propagation or stored at −80°C. After six blind passages, culture supernatants were collected and used for further investigation.

### Plaque purification of PEDV.

Vero cells were infected in the presence of trypsin with serial passages from virus stocks as reported previously (29). Briefly, the culture supernatants were collected at either 24 or 48 h postinfection (hpi) according to when 80% CPEs were observed. Vero cells in 12-well plates were inoculated with 100 μL of 10-fold serially diluted culture supernatants. After 1 h of adsorption at 37°C, the cell monolayers were washed with PBS, and then they were overlaid with DMEM containing 1% low-melting-point agarose and 15 μg/mL trypsin. After solidification of the gel overlay, the plates were inverted and then they were placed in an incubator at 37°C with 5% CO_2_. At 3 to 4 days postinfection (dpi), either the plaques were selected for cell infection or they were visualized after crystal violet staining. The PEDV isolates of CH/GXNN-1/2018 were plaque purified serially three times, and the purified viruses were subjected to serial passages and then they were measured using RT-PCR.

### Indirect immunofluorescence assay (IFA).

Vero cells in 6-well culture plates were infected with PEDVs at a multiplicity of infection (MOI) of 0.01. After 72 h, the cells were washed twice with PBS containing 0.05% Tween-20 (PBST), and the cells were fixed with cold formaldehyde and blocked with 1% bovine serum albumin (BSA). They were then incubated at 37°C for 2 h with 1: 2,000 diluted anti-PEDV S protein monoclonal antibody (Median, Chuncheon, South Korea). After a wash step with PBST, a 1:5,000 diluted Alexa Fluor 488 conjugated goat anti-mouse IgG (H+L) antibody (Abcam, UK) was added, and then the cells were further incubated at 37°C for 1 h. The cells were then incubated with 4′,6-diamidino-2-phenylindole (DAPI; Beyotime). Finally, the cells were washed, and then they were observed under a fluorescence microscope (Nikon, Japan).

### Electron microscopy assay.

Vero cells were infected with PEDVs, and the cell cultures were collected when CPEs were observed to exceed 80%. The samples were frozen and thawed at −80°C three times and then centrifuged at 10,000 rpm at 4°C for 1 h. The collected cell supernatants were filtered through a 0.22-μm filter. Then they were mixed with 50% polyethylene glycol 8000 (PEG-8000) to a final concentration of 10%, precipitated, and stirred gently at 4°C overnight. After a 12,000-rpm centrifugation step at 4°C for 2 h, the precipitated viruses were resuspended in Tris-buffered saline (TBS) solution. Then the cells were negatively stained with 2% phosphotungstic acid, and they were imaged with a transmission electron microscope (TEM) system (Hitachi, Japan).

### Fluorescence quantitative PCR.

Virus supernatants were collected and cDNA was prepared as mentioned previously. The determination of virus copy number of 12 isolates in Vero cells was performed by using fluorescence quantitative PCR using TB Green premix *Ex Taq* II (TaKaRa, Japan). Sequence primers targeting the M gene were designed, and they were qPEDV-MF (5′-GGAATTTCACATGGAATATCA-3′) and qPEDV-MR (5′-CCATAGAAT AGCCATCTTGAC-3′).

### Multistep growth curves of viruses.

For growth curve analysis, Vero cells in 12-well plates were inoculated with PEDVs at an MOI of 0.001 or 0.01. After adsorption at 37°C for 1 h, followed by two washes with PBS, the supernatants and the infected cells were collected at 6, 12, 24, 36, 48, and 60 hpi and stored at −80°C for virus titer determination. The virus titers in Vero cells at each time point were determined in triplicate by using plaque assays.

### S genetic evolutionary tree construction and gene sequence analysis.

Viral RNA extraction and cDNA preparation were performed as described above. The primers used for the full sequence amplification of the S gene in the 12 samples (32) were PED-SF (5′-ATTGTAAGCATAAGGCTACAG-3′) and PED-SR (5′-GAAGCTTGTCTAATTGGAACT-3′). The primers used for the amplification of the ORF3 gene were PED-ORF3F (5′-GTCCTAGACTTCAACCTTACGAAG-3′) and PED-ORF3R (5′-AACTACTAGACCATTATCATTCAC-3′) (33). A total of 118 sequences from the representative PEDV strains from various countries and additional sequences were downloaded from GenBank (data not shown) and subjected to phylogenetic analysis. Phylogenetic trees were constructed using the neighbor-joining method and MEGA 6.0, with bootstrap values calculated for each node from 1,000 replicates. The resulting tree was visualized using Interactive Tree of Life (iTOL) v.6 (http://itol.embl.de/, accessed on 20 April 2022). A comparison of the amino acid sequences of the S and ORF3 genes was analyzed using the Molecular Evolutionary Genetics Analysis software Megalign.

### Pig challenge experiment.

Eight 7-day-old piglets were purchased from a commercial pig farm with no prior PEDV vaccination program and no history of PED. All piglets were diagnosed as negative for PEDV, transmissible gastroenteritis virus (TGEV), and rotavirus (PoRV) by using a virus-specific RT-PCR analysis of rectal swabs. They were also determined to be free of antibodies to PEDV by using a commercial PEDV antibody enzyme-linked immunosorbent assay (ELISA) kit (Biovet Inc, St-Hyacinthe, QC, Canada), according to the manufacturer’s instructions. All piglets were divided randomly into 2 groups and they were housed in separate animal rooms. Group 1 (*n* = 4) was inoculated orally with 2 mL of CH/GXNN-1/2018 virus solution (10^6^ PFU/mL) and group 2 (*n* = 4) was inoculated orally with 2 mL of cell culture medium. Clinical signs for each group were recorded every 12 h. During the experiment, the piglets had their body temperatures and weights measured regularly and were monitored for clinical signs of disease, including appetite status, mental status, hair condition, diarrhea, and vomiting. The following criteria were used for the assessment of animals during the experiment: 0 for normal, 1 for slight change, 2 for obvious change, and 3 for severe change (17). Rectal swabs were collected at different time points after PEDV infection for scoring fecal denseness (34) and were placed in Eppendorf (EP) tubes containing 4 mL of PBS for the measurement of fecal viral RNA shedding by RT-qPCR as described above. All surviving piglets from the challenged and control groups were euthanized at 5 dpi for postmortem examinations. The piglets were dissected; the stomachs, duodenum, jejunum, ileum, cecum, and mesenteric lymph nodes were fixed in 4% paraformaldehyde and stained with hematoxylin and eosin (H&E) and subjected to immunohistochemistry (IHC) in order to detect viruses in the tissues.

Veterinary personnel were involved throughout the study, and all the necessary protective measures were followed to ensure minimal animal suffering. The animal care and procedures used in this study were carried out in strict accordance with Guangxi University Animal Care and Welfare (number GXU2020-022).

### H&E and IHC staining.

Samples of the duodenum, jejunum, ileum, cecum, mesenteric lymph nodes (MLN), and stomach were fixed in 4% paraformaldehyde for H&E staining. The anti-PEDV S protein monoclonal antibody (Median, Chuncheon, South Korea; diluted 1:2,000) and horseradish peroxidase (HRP)-conjugated goat anti-mouse IgG (H+L) antibody (Servicebio, Wuhan, China; diluted 1:200) were used for IHC staining. The results were observed by light microscopy (Eclipse Ci; Nikon, Japan) and photographed by using an imaging system (digital sight DS-FI2; Nikon, Japan).

### Virus neutralization (VN) test.

Serum samples from all piglets (*n* = 8) collected at 0, 24, 72, and 120 hpi were tested for PEDV neutralizing antibodies against the CH/GXNN-1/2018 strain inoculated in this study and the G2b strain 17GXCZ-1ORF3d (29). Briefly, 2-fold serial dilutions of serum were coincubated with equal volumes of viral stock containing 200 TCID_50_ of virus at 37°C for 1 h. Then, the mixture was inoculated into the Vero cell monolayers in 96-well tissue culture plate, and the plate was washed with PBS and incubated at 37°C for 1 h. After incubation, the mixture was discarded, and the plate was washed again with PBS. Next, a maintenance medium containing trypsin (15 μg/mL) was added to each well, and the plate was incubated for 5 days at 37°C. Neutralizing antibody titers were calculated as the highest serum dilution that completely inhibited CPEs.

### Statistical analysis.

Statistical significance between the control and the infection groups was determined by using one-way analysis of variance (ANOVA). Differences were considered significant when the *P* value was less than 0.05.

### Ethical statement.

The animal study was reviewed and approved by the Animal Care & Welfare Committee of Guangxi University (no: GXU2020-022).

### Data availability.

The complete S genome sequences of 12 PEDV strains obtained in this study have been deposited in the GenBank under the accession numbers MZ669834-MZ6698345.
